# Melatonin Rescues Heat Stress-Induced Suppression of TCA Cycle and Mitochondrial Damage in Goat Sertoli Cells

**DOI:** 10.3390/ijms262311475

**Published:** 2025-11-27

**Authors:** Guang Yang, Yilin Shan, Zhen Zhang, Hao Wu, Yuanyuan Lei, Yichong Sun, Pengyun Ji, Liu Guoshi

**Affiliations:** 1 College of Animal Science and Technology, Sanya Institute of China Agricultural University, Sanya 572025, China; yangguangimu@163.com (G.Y.); 18749839229@163.com (Y.S.); zhangzhencarl@163.com (Z.Z.); lyy2916684423@163.com (Y.L.); sunyichong2021@126.com (Y.S.); jipengyun@cau.edu.cn (P.J.); 2 State Key Laboratory of Farm Animal Biotech Breeding, Frontiers Science Center for Molecular Design Breeding, College of Animal Science and Technology, China Agricultural University, Beijing 100193, China; 18800160525@163.com

**Keywords:** heat stress, goat, Sertoli cells, melatonin, metabolic disorder, metabolic flux analysis, transcriptomics

## Abstract

Global climate change exacerbates heat stress, a major threat to male fertility in livestock. As Sertoli cells (SCs) are essential for spermatogenesis but are highly vulnerable to heat, understanding the underlying metabolic dysfunction is critical. This study investigated heat-induced metabolic dysregulation in goat SCs and evaluated the protective effects of melatonin. Through integrated transcriptomics and stable isotope-assisted metabolic flux analysis (MFA), we mapped alterations in gene expression and central carbon metabolism. Heat stress provoked mitochondrial dysfunction and oxidative damage. Metabolic flux analysis revealed suppressed mitochondrial oxidative metabolism, evidenced by reduced glucose-derived carbon incorporation into TCA cycle intermediates. Transcriptomics identified key perturbations in energy metabolism and sphingolipid signaling. Melatonin pretreatment restored mitochondrial function and redox homeostasis, and triggered a protective metabolic reprogramming that enhanced cell viability. Our findings demonstrate that melatonin mitigates heat stress by preserving mitochondrial integrity and redox homeostasis, providing mechanistic insights into male infertility and suggesting melatonin as a promising therapeutic for enhancing livestock reproductive resilience under climate stress.

## 1. Introduction

Global climate change, characterized by the increasing frequency, intensity, and duration of heatwaves, poses a significant threat to the sustainability of animal husbandry worldwide [1,2]. Heat stress has emerged as a critical factor impairing male reproductive performance in livestock, particularly in economically important species such as goats [3,4]. Spermatogenesis is highly sensitive to elevated testicular temperatures (typically 2–4 °C below core body temperature) [5,6], rendering male reproduction particularly vulnerable to climate change [7,8]. Seasonal high temperatures adversely impact semen quality and breeding efficiency, underscoring the urgent need to elucidate the physiological mechanisms underlying heat stress-induced male infertility to develop effective mitigation strategies [9,10].

Within the testicular microenvironment, Sertoli cells (SCs) serve as indispensable support structures for developing germ cells, providing essential structural, nutritional, and metabolic support [11,12]. A defining feature of SCs is their role in forming and maintaining the blood-testis barrier (BTB), which establishes an immunologically privileged microenvironment essential for meiosis and spermiogenesis [13,14]. Crucially, SCs exhibit a Warburg-like metabolism, characterized by high glycolytic flux and lactate production, to supply energy substrates for germ cells [15,16]. We hypothesized that under heat stress, this specialized metabolic state is pathologically exacerbated, leading to a severe suppression of mitochondrial oxidative metabolism. We further postulated that such metabolic disruption would result in ER stress, oxidative damage, and ultimately compromise BTB integrity and trigger apoptosis. Supporting this, recent research on goats suggests that heat stress triggers SC apoptosis via an ROS-DRP1-mediated pathway [3], resulting in excessive mitochondrial fission and impaired mitophagy, thereby compromising spermatogenic efficiency.

Metabolic disruption is central to how heat stress impairs SC function [17,18]. Heat stress significantly alters key cellular metabolic pathways [18,19]. Such disruption is likely to impair cellular energy supply and redox homeostasis, particularly through diminished NADPH production, contributing to oxidative damage accumulation [20,21]. However, a critical gap persists in our understanding of the dynamic metabolic alterations induced by heat stress. Conventional transcriptomic and metabolomic approaches, while valuable for identifying changes in gene expression and metabolite abundance, are inherently static in nature. These methods capture snapshots of cellular states at a single time point, but they cannot reveal the active flow of metabolites through biochemical pathways—the true functional readout of metabolic activity. This limitation is particularly relevant in the context of heat stress, where rapid and adaptive remodeling of carbon flux is expected. Key questions, such as the extent of TCA cycle suppression and its functional consequences on energy metabolism and redox balance, remain unaddressed by static ‘omics’ data alone.

In recent years, melatonin has garnered significant attention for its protective roles against various stressors, including heat stress [22]. As a potent antioxidant and regulator of circadian rhythms, melatonin has been demonstrated to mitigate oxidative damage, modulate metabolic pathways, and enhance cellular resilience under stressful conditions. Its ability to alleviate heat stress-induced impairments in reproductive function has been increasingly reported, highlighting its potential as a therapeutic agent in livestock production [23,24]. However, the specific mechanisms by which melatonin modulates metabolic flux and restores redox homeostasis in SCs under heat stress remain poorly understood.

To overcome these limitations, the present study employed an integrated approach, combining transcriptomics with stable isotope-assisted metabolic flux analysis (MFA). Unlike static analyses, MFA utilizes tracer compounds such as ^13^C-labeled glucose (^13^C_6_) to dynamically track the distribution of carbon atoms through metabolic networks. This enables direct quantification of pathway fluxes—such as glycolytic throughput, pentose phosphate pathway activity, and TCA cycle turnover—providing a functional measure of metabolic phenotype that is inaccessible via conventional omics. This methodological approach is necessary to accurately capture the real-time metabolic reprogramming in SCs under heat stress. Furthermore, we introduced melatonin—a potent antioxidant and neurohormone known to alleviate heat stress-induced reproductive damage—as an interventional tool. We hypothesized that heat stress induces metabolic disorders in SCs, characterized by dysregulation of the TCA cycle, which collectively disrupts energy and redox homeostasis. We further hypothesized that melatonin exerts cytoprotective effects by normalizing these metabolic alterations.

This study aimed to delineate the metabolic disorders in goat SCs under heat stress. Furthermore, we sought to elucidate the protective mechanism of melatonin by integrating transcriptomic and metabolic flux analyses. Our findings are expected to provide novel insights into the pathophysiology of heat-induced male infertility and to identify potential metabolic targets for therapeutic interventions in livestock production systems facing the challenges of climate change.

## 2. Results

### 2.1. Melatonin Preserves Viability of Heat-Stressed Goat Sertoli Cells

**Figure 1:**
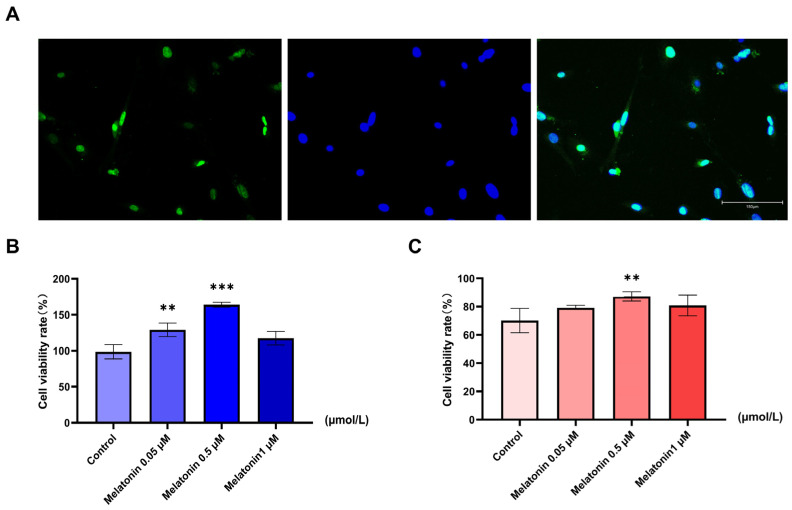
Identification of goat SCs and the effects of melatonin on cell viability under normal and heat stress conditions. (**A**) Identification of SCs by SOX9 immunofluorescence (green) and DAPI (blue) staining (scale bar = 150 µm). (**B**) Viability of SCs treated with melatonin (0–1 µM) under normal conditions (37 °C). (**C**) Viability of SCs pretreated with melatonin (0–1 µM) followed by heat stress (42 °C, 0.5 h). Data are mean ± SD from three independent experiments (*n* = 3). ** *p* < 0.01, *** *p* < 0.001 vs. control.

Goat SCs were successfully isolated, as confirmed by positive immunofluorescence staining for the specific marker SOX9 (Figure 1A). Under normal culture conditions (37 °C), melatonin treatment (0.5 µM) significantly enhanced cell viability compared to the vehicle control (*p* < 0.001) (Figure 1B). Exposure to heat stress (42 °C for 0.5 h) markedly reduced cell viability, and this reduction was significantly attenuated by pretreatment with 0.5 µM melatonin (*p* < 0.01) (Figure 1C). These results identified 0.5 µM as the optimal concentration of melatonin for protecting SC viability against heat stress, and this concentration was used in all subsequent experiments.

### 2.2. Transcriptomic Profiling of Heat-Stressed SCs

**Figure 2:**
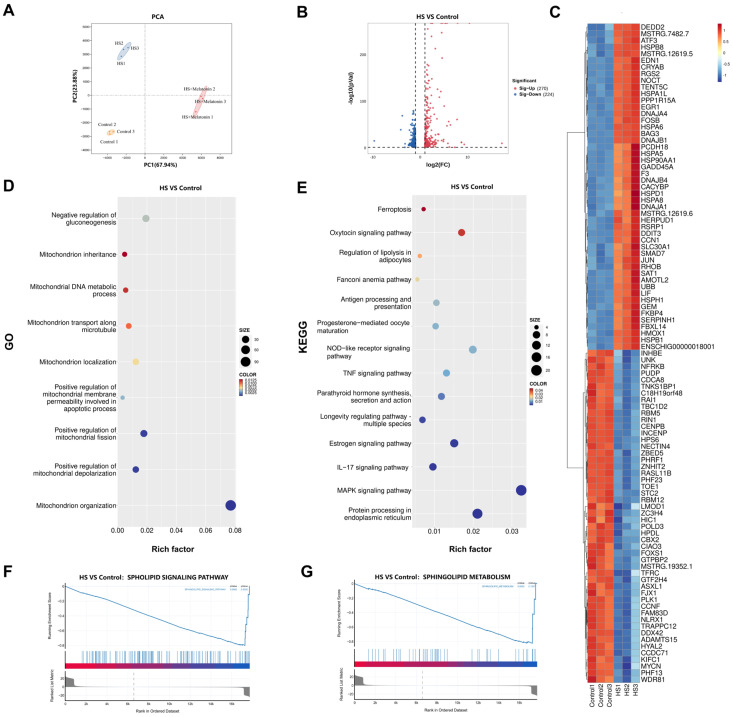
Transcriptomic alterations in goat SCs induced by heat stress. (**A**) PCA of transcriptomes from Control, HS, and HS + Melatonin groups (*n* = 3 biologically independent samples per group). (**B**) Volcano plot of DEGs between Control and HS. (**C**) Heatmap of the top 50 up- and down-regulated DEGs. (**D**) A focused view of GO terms related to mitochondrial function. (**E**) Top 10 enriched KEGG pathways. (**F**,**G**) GSEA enrichment plots for the (**F**) SPHINGOLIPID SIGNALING PATHWAY and (**G**) SPHINGOLIPID METABOLISM.

We performed RNA sequencing on goat SCs under control, HS, and HS + melatonin conditions. Principal component analysis (PCA) revealed a clear separation between the Control and HS groups along PC1 (Figure 2A). Analysis identified 494 differentially expressed genes (DEGs) between Control and HS, with 270 upregulated and 224 downregulated (Figure 2B). Unsupervised hierarchical clustering of the top 50 differentially expressed genes (DEGs) clearly segregated the experimental groups (Figure 2C). Gene Ontology (GO) term analysis revealed significant enrichment in broad biological processes. A focused examination specifically highlighted pronounced alterations in mitochondrial organization, transport, and membrane permeability (Figure 2D). Kyoto Encyclopedia of Genes and Genomes (KEGG) pathway analysis revealed top enriched pathways such as Ferroptosis and the NOD-like receptor signaling pathway (Figure 2E). Furthermore, Gene Set Enrichment Analysis (GSEA) confirmed significant enrichment in the sphingolipid signaling and metabolism pathways (Figure 2F,G). These results demonstrate that heat stress induces significant alterations in the transcriptional landscape, with a significant impact on mitochondrial function and sphingolipid metabolism.

### 2.3. Melatonin Reverses Heat Stress-Induced Transcriptional Alterations in Metabolic Pathways

**Figure 3:**
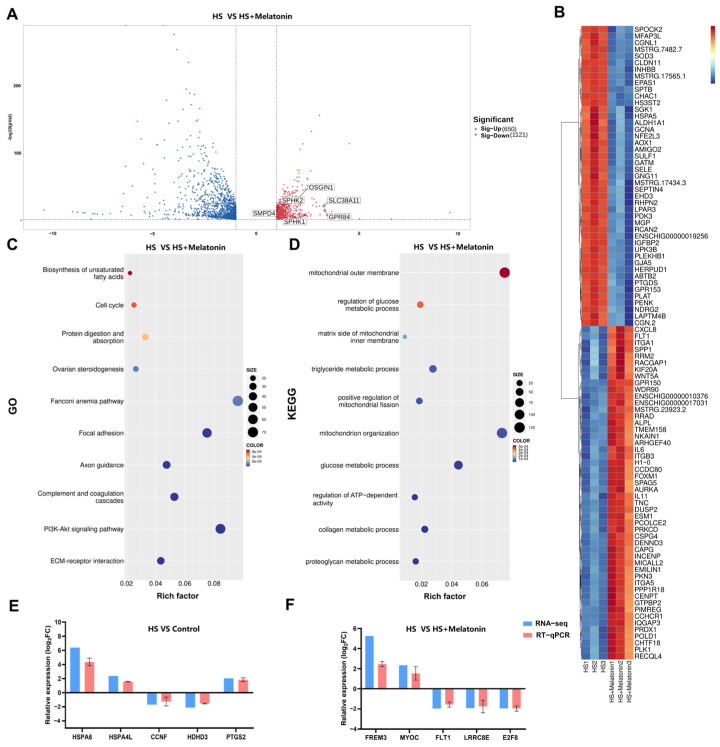
Melatonin attenuates heat stress-induced metabolic alterations in goat testicular SCs. (**A**) Volcano plot of DEGs between HS + Melatonin and HS groups (650 upregulated, 1121 downregulated). Selected energy metabolism genes are highlighted. (**B**) Heatmap of the top 50 up- and down-regulated DEGs. (**C**) A focused view of GO terms related to mitochondrial function. (**D**) Top 10 enriched KEGG pathways. (**E**,**F**) RT-qPCR validation of ten selected DEGs. Data represent mean ± SD from three independent experiments (*n* = 3).

Having established the transcriptomic alterations induced by heat stress, we next sought to determine how melatonin mitigates these changes. To elucidate the transcriptional mechanisms underlying melatonin’s protective effects, we compared the transcriptomes of the HS + Melatonin and HS groups, which identified 650 upregulated and 1121 downregulated DEGs (Figure 3A). Notably, melatonin upregulated genes involved in multiple cytoprotective processes, including antioxidant response (e.g., *OSGIN1* [25,26], which enhances cellular defense via the Nrf2 pathway), sphingolipid metabolism (e.g., *SPHK1* [27,28] and *SPHK2* [29,30], responsible for phosphorylating sphingosine to generate sphingosine-1-phosphate, and *SMPD4* [31]), lipid metabolism (e.g., *GPR84* [32]), and amino acid transport (e.g., *SLC38A11* [33]). Unsupervised clustering of the top 50 DEGs clearly distinguished the treatment groups (Figure 3B), reinforcing the distinct transcriptomic profiles associated with each condition. We then performed a manual screening of the GO enrichment results, which confirmed a significant overrepresentation of terms specifically related to mitochondrial metabolism (Figure 3C). In parallel, KEGG pathway analysis highlighted several metabolic pathways modulated by melatonin, such as the “Biosynthesis of unsaturated fatty acids” (Figure 3D). To experimentally validate the RNA-seq findings, RT-qPCR was performed on ten representative DEGs across the Control, HS, and HS + Melatonin groups. The expression patterns obtained by RT-qPCR were consistent with the transcriptomic data (Figure 3E,F), confirming the reliability of our sequencing results.

### 2.4. Melatonin Preserves Mitochondrial Ultrastructure and Function in Heat-Stressed SCs

**Figure 4:**
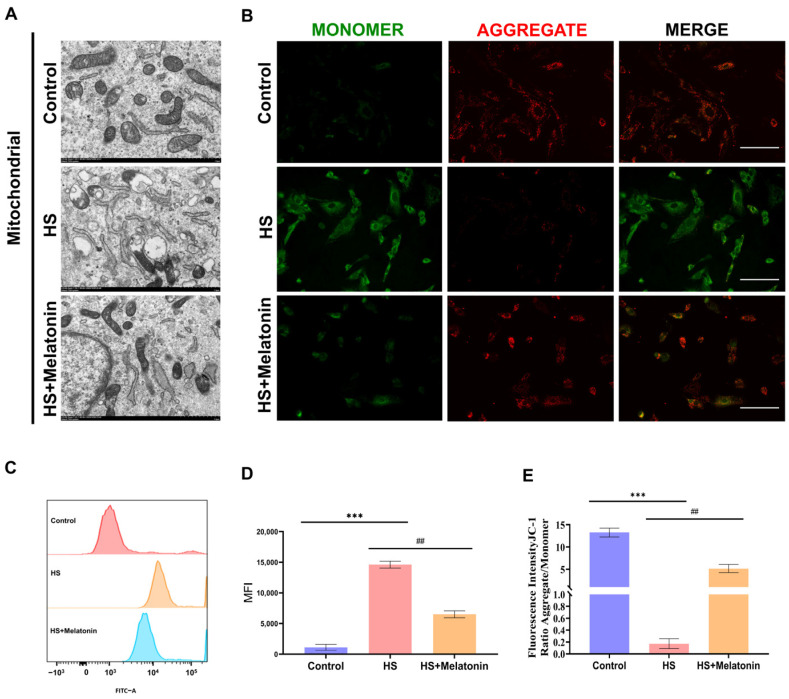
Melatonin preserves mitochondrial structure and function in heat-stressed goat SCs. (**A**) Representative TEM images of mitochondrial ultrastructure (images are representative of three independent experiments; *n* = 3) (scale bar = 1 μm). (**B**) JC-1 staining showing mitochondrial membrane potential (images are representative of three independent experiments; *n* = 3) (red: J-aggregates, high ΔΨm; green: J-monomers, low ΔΨm) (scale bar = 150 μm). (**C**) Representative flow cytometry histograms of MitoSOX Red fluorescence, analyzed with FlowJo, indicating mitochondrial superoxide levels (histograms are representative of three independent experiments; *n* = 3). (**D**,**E**) Quantification of MitoSOX fluorescence intensity and JC-1 red/green ratio. Data are mean ± SD from three independent experiments (*n* = 3); *** *p* < 0.001 vs. Control; ## *p* < 0.01.

We next assessed whether the metabolic and transcriptional alterations induced by heat stress were associated with structural and functional damage to mitochondria. Transmission Electron Microscopy (TEM) revealed that heat stress severely disrupted mitochondrial ultrastructure, characterized by cristae fragmentation and outer membrane rupture in SCs (Figure 4A). In contrast, pretreatment with melatonin markedly preserved mitochondrial integrity, with a higher proportion of organelles exhibiting intact double membranes and organized cristae (Figure 4A). To evaluate functional consequences, we examined the mitochondrial membrane potential (ΔΨm) using JC-1 staining. Heat stress significantly dissipated ΔΨm, as indicated by a decreased red-to-green fluorescence ratio compared to the control group (*p* < 0.001). Notably, melatonin pretreatment significantly, though partially, restored ΔΨm (*p* < 0.01 vs. HS group) (Figure 4B,E). We further measured mitochondrial superoxide levels using MitoSOX™ Red. Heat stress induced a pronounced increase in mitochondrial ROS (*p* < 0.001 vs. control), which was significantly attenuated by melatonin treatment (*p* < 0.01 vs. HS) (Figure 4C,D). Collectively, these results demonstrate that heat stress induces severe mitochondrial damage and functional impairment in goat SCs, while melatonin effectively preserves mitochondrial structure, membrane potential, and redox homeostasis.

### 2.5. Melatonin Orchestrates a Protective Metabolic Reprogramming Beyond Simple Reversal of Heat Stress Effects

**Figure 5:**
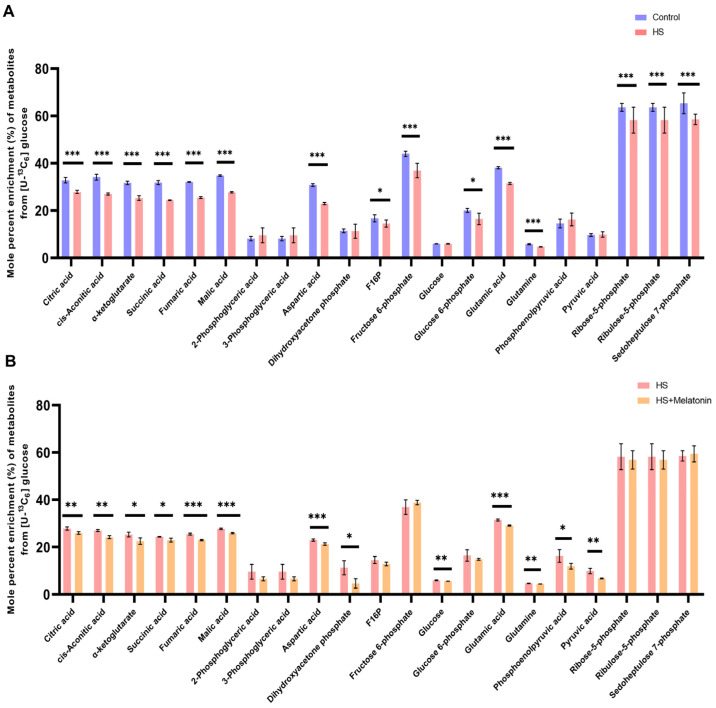
Melatonin reprograms central carbon metabolism under heat stress. (**A**) Comparison between Control and HS groups: MPE of selected metabolites from [U-^13^C_6_] glucose in central carbon metabolism pathways. (**B**) Comparison between HS and HS + Melatonin groups: Melatonin pretreatment does not reverse but further attenuates the MPE of TCA cycle intermediates and glycolytic precursors. Data are presented as mean ± SD from three independent experiments (*n* = 3). * *p* < 0.05, ** *p* < 0.01, *** *p* < 0.001. M0 = unlabeled metabolite (no ^13^C incorporated); M + *n* = isotopologue containing *n* labeled carbon atoms.

To quantitatively assess the impact of heat stress on mitochondrial metabolic function and the potential protective role of melatonin, we conducted stable isotope tracing using [U-^13^C_6_] glucose in SCs. Analysis of the ^13^C-labeling patterns demonstrated that heat stress markedly disrupted mitochondrial metabolic pathways relative to the control (Figure 5A). MFA revealed significant alterations in central carbon metabolism under heat stress. A pronounced reduction in the mole percent enrichment (MPE) was observed for all major TCA cycle intermediates—citrate, cis-aconitate, α-ketoglutarate, succinate, fumarate, and malate (*p* < 0.001). Similarly, the pentose phosphate pathway was impaired, with significantly decreased enrichment in ribose-5-phosphate, ribulose-5-phosphate, and sedoheptulose-7-phosphate (*p* < 0.001). Consequently, the labeling of amino acids derived from these pathways—aspartate, glutamate, and glutamine—was substantially lower in heat-stressed cells (*p* < 0.001). In contrast, the enrichment of glycolytic intermediates, including 2-phosphoglycerate and 3-phosphoglycerate, remained unchanged (Figure 5A). These data collectively demonstrate that acute heat stress severely suppresses glucose oxidation in the mitochondrial TCA cycle.

Unexpectedly, in the HS + Melatonin group, MFA revealed that melatonin did not reverse the heat stress-induced suppression of glucose oxidation. On the contrary, it led to a further reduction in the MPE of TCA cycle intermediates derived from glucose, including citrate, cis-aconitate, fumarate, and malate (*p* < 0.01), as well as in the glycolytic precursors (Figure 5B). This counterintuitive finding indicates that melatonin’s potent cytoprotection is not mediated by a simple restoration of the pre-stress metabolic flux but is instead associated with a comprehensive metabolic reprogramming that actively shifts carbon utilization away from mitochondrial glucose oxidation.

### 2.6. Melatonin Further Attenuates Glucose-Derived Carbon Flux into the TCA Cycle Under Heat Stress

**Figure 6:**
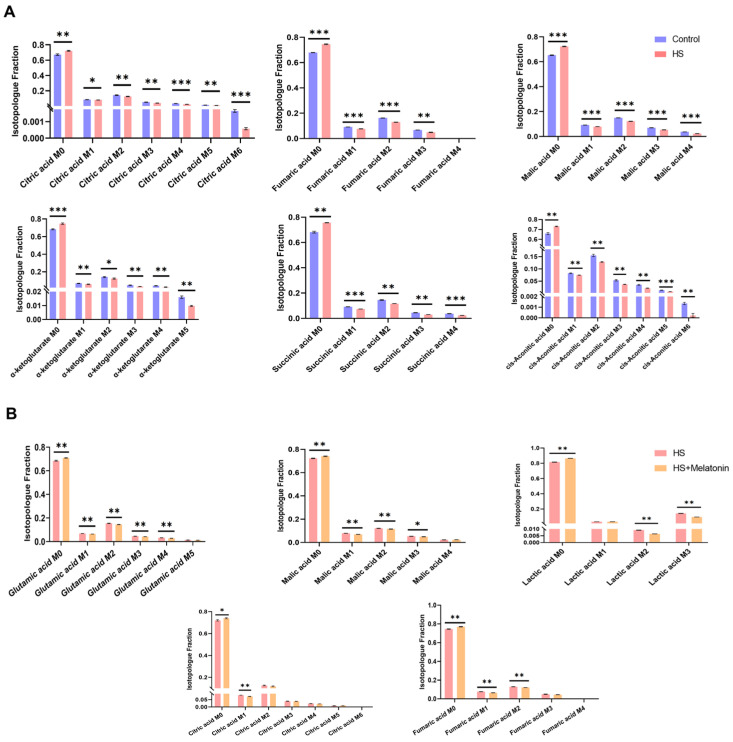
Isotopologue fraction analysis of TCA cycle intermediates in goat SCs. (**A**) Comparison between Control (37 °C, 0.1% DMSO) and HS (42 °C for 0.5 h, 0.1% DMSO) groups. (**B**) Comparison between HS and HS + Melatonin (42 °C for 0.5 h, 0.5 µM melatonin) groups. Data are mean ± SD from three independent experiments (*n* = 3). * *p* < 0.05, ** *p* < 0.01, *** *p* < 0.001. M0 = unlabeled metabolite (no ^13^C incorporated); M + *n* = isotopologue containing *n* labeled carbon atoms.

Mass isotopomer distributions (MIDs) analysis from our [U-^13^C_6_] glucose tracing experiment revealed that heat stress significantly altered the TCA cycle metabolism in goat SCs (Figure 6A). Compared to the Control, HS cells exhibited a consistent increase in the unlabeled (M0) fraction, accompanied by a decrease in the labeled (M + 1 to M + *n*) isotopologues across all detected intermediates, including citrate, fumarate, malate, succinate, cis-aconitate, and α-ketoglutarate, indicating a marked attenuation of glucose-derived carbon influx into the TCA cycle.

Melatonin further reduced the labeling fractions of most metabolites under heat stress (Figure 6B), indicating decreased utilization of external glucose for anabolic processes. Consistent with the MPE data, these MID results reinforce that melatonin’s protection is coupled with a diverted flux of glucose carbon away from mitochondrial oxidation.

### 2.7. Melatonin Restores Mitochondrial Respiratory Function Despite Reduced Glucose Oxidation

**Figure 7:**
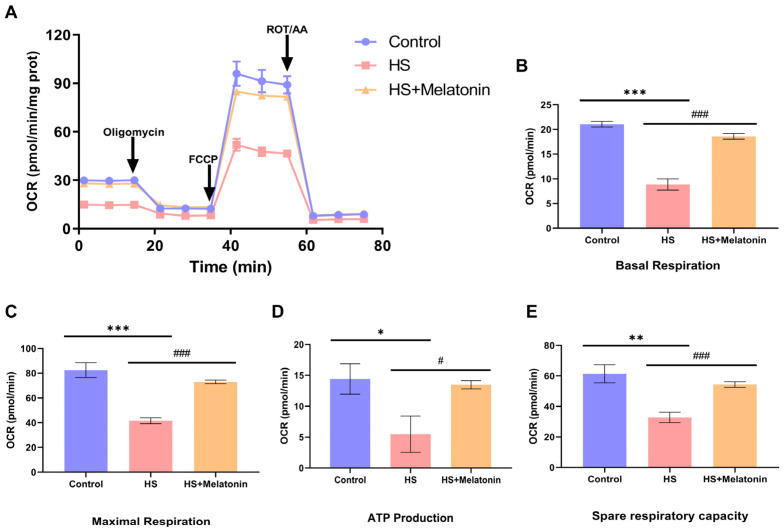
Melatonin restores mitochondrial respiratory function in heat-stressed goat SCs. (**A**) Representative OCR traces in response to sequential injection of oligomycin, carbonyl cyanide-p-trifluoromethoxyphenylhydrazone (FCCP), and a mix of rotenone and antimycin A (Rot/AA). (**B**–**E**) Quantification of (**B**) basal respiration, (**C**) maximal respiration, (**D**) ATP-linked production, and (**E**) spare respiratory capacity. Cells were treated as follows: Control (37 °C), heat stress (HS, 42 °C for 0.5 h), and HS + Melatonin (0.5 µM melatonin pretreatment + 42 °C for 0.5 h). All media contained 0.1% DMSO. Data are presented as mean ± SD (*n* = 3); * *p* < 0.05, ** *p* < 0.01, *** *p* < 0.001 vs. Control group; # *p* < 0.05, ### *p* < 0.001 vs. HS group.

We next sought to resolve the apparent paradox between the reduced incorporation of glucose-derived carbon into the TCA cycle (Figure 5 and Figure 6) and the preservation of mitochondrial ultrastructure and membrane potential (Figure 4) in melatonin-pretreated cells. To this end, we assessed the functional output of mitochondrial metabolism by directly measuring the oxygen consumption rate (OCR). Strikingly, despite the further attenuation of glucose oxidation, melatonin pretreatment significantly restored mitochondrial respiratory function in heat-stressed SCs (Figure 7A).

Quantitative analysis of key parameters demonstrated that heat stress significantly suppressed basal respiration (Figure 7B), maximal respiration (Figure 7C), and ATP production (Figure 7D) compared to the control. Pretreatment with melatonin (HS + Melatonin) significantly attenuated these deficits, restoring all measured parameters toward the levels observed in the control group (Figure 7B–E).

These results provide direct functional evidence that the restoration of mitochondrial bioenergetics by melatonin occurs independently of reinstating glucose oxidation. This critical finding corroborates the metabolic reprogramming suggested by our MFA data and indicates that melatonin enables heat-stressed SCs to maintain energy production by utilizing alternative substrates to fuel oxidative phosphorylation.

## 3. Discussion

Heat stress is a major cause of male infertility, primarily by disrupting testicular cell metabolism—a central, yet incompletely elucidated, pathogenic mechanism [34]. In Sertoli cells, germ, and Leydig cells, this metabolic dysregulation disrupts nutrient transport, energy production, and signal transduction, culminating in spermatogenic failure and compromised sperm quality [35,36]. Heat stress also induces significant oxidative stress, marked by elevated ROS and MDA levels, which damages sperm DNA, promotes apoptosis, and increases abnormal sperm production [37,38]. However, the molecular mechanisms and acute functional impact of these metabolic alterations remain unclear, impeding the development of targeted interventions [39,40].

In this study, we combined transcriptomics with stable isotope-based MFA in goat Sertoli cells to investigate heat stress-induced metabolic changes. Our results establish the suppression of the mitochondrial TCA cycle as a critical event in heat stress-induced dysfunction. MFA provided direct evidence of a severe reduction in glucose-derived carbon flux into the TCA cycle. Transcriptomic profiling identified 494 DEGs in heat-stressed Sertoli cells, with significant enrichment in mitochondrial organization, membrane permeability, and sphingolipid metabolism—highlighting the central role of mitochondrial dysfunction. Enrichment of ferroptosis and NOD-like receptor signaling pathways further indicated that oxidative damage and inflammatory activation contribute to Sertoli cell impairment [40,41].

Notably, melatonin treatment effectively reversed these alterations. Upregulation of cytoprotective genes—such as *OSGIN1* (linked to Nrf2-mediated antioxidant response) [26], *SPHK1/2* (involved in sphingolipid metabolism), and genes related to lipid and amino acid transport—underscore melatonin’s multifaceted protective mechanisms [29,42]. This restorative effect was further supported by the enrichment of mitochondrial metabolism-related GO terms and KEGG pathways like “Biosynthesis of unsaturated fatty acids.” RT-qPCR validation confirmed that melatonin alleviates heat stress-induced metabolic dysregulation primarily by restoring transcriptional homeostasis, particularly in mitochondrial integrity and sphingolipid signaling.

Our integrated analysis reveals that melatonin’s protection extends beyond simple metabolic restoration. Transcriptomic data show that melatonin upregulates genes involved in antioxidant defense, sphingolipid metabolism, lipid metabolism, and amino acid transport, suggesting a coordinated shift in cellular metabolism [43,44]. Interestingly, MFA revealed that melatonin did not reverse the heat-induced suppression of glucose oxidation in the TCA cycle; instead, it further reduced the MPE of TCA intermediates such as citrate, cis-aconitate, fumarate, and malate.

Our study reveals a compelling paradox: melatonin restores mitochondrial respiration without concomitantly reinstating glucose oxidation. This finding strongly suggests that melatonin induces a strategic metabolic reprogramming in heat-stressed SCs. Upregulation of sphingolipid and lipid metabolic genes, along with enrichment of unsaturated fatty acid biosynthesis, implies a shift toward alternative energy substrates. Specifically, the transcriptomic upregulation of lipid metabolism gene GPR84 and amino acid transporter gene SLC38A11 (as described in Section 2.3) provides genetic evidence for enhanced utilization of non-carbohydrate substrates, indirectly supporting the carbon flux redistribution away from glucose and toward fatty acids or amino acids to fuel mitochondrial respiration. Critically, the role of melatonin in further oxidation extends beyond simply providing alternative substrates; it orchestrates a broader metabolic rewiring that supports redox homeostasis and sustains mitochondrial respiration under stress. By enhancing the oxidation of lipids and possibly amino acids, melatonin may help maintain NAD + /NADH balance and reduce electron leakage, thereby limiting ROS generation from impaired glucose oxidation [45,46]. We propose that under melatonin’s influence, heat-stressed Sertoli cells prioritize fatty acids or amino acids over glucose to fuel the TCA cycle and oxidative phosphorylation [47]. Reduced glucose carbon flux may reflect rerouting of glycolytic intermediates toward biosynthetic or redox-balancing pathways, such as the pentose phosphate pathway [48,49]. Thus, melatonin acts not merely as an antioxidant, but as a key regulator of metabolic flexibility, enabling adaptive reprogramming to preserve mitochondrial function under stress.

A major strength of our study is the use of stable isotope-based MFA, which offers dynamic, quantitative insights into central carbon metabolism that are inaccessible through conventional methods [50,51]. While mitochondrial respiration assays confirmed TCA cycle impairment under heat stress, MFA precisely identified the defect: reduced incorporation of glucose-derived carbon into the cycle, reflected by increased unlabeled (M0) fractions and decreased labeled isotopologues of key intermediates. This approach provides a deeper understanding than static metabolite measurements [18].

Although oxidative stress and apoptosis are well-established consequences of heat stress [52,53]. our integrated analysis positions metabolic dysregulation as an early and central pathogenic event, potentially upstream of redox imbalance and cell death. We directly observed acute TCA cycle suppression via MFA, alongside transcriptomic changes in mitochondrial and sphingolipid pathways, reinforcing the primacy of metabolic disruption.

Moreover, melatonin’s cytoprotective effects go beyond its antioxidant role. While it restored mitochondrial respiration, MFA showed that this recovery occurred without reinstating glucose-derived carbon influx into the TCA cycle. This dissociation between bioenergetic recovery and glucose oxidation highlights melatonin’s role in metabolic reprogramming. We propose that melatonin enhances metabolic flexibility in heat-stressed Sertoli cells, shifting energy substrate utilization from glucose toward alternatives such as fatty acids. Although our tracing data do not directly identify the alternative fuels, future studies using parallel isotope tracing from fatty acids or glutamine could test this hypothesis.

In summary, our integrated transcriptomic and metabolic flux analyses provide a comprehensive perspective on how heat stress impairs Sertoli cell function and how melatonin counteracts these effects. Melatonin’s ability to restore mitochondrial metabolism and enhance antioxidant defenses underscores its potential as a therapeutic agent against heat-induced reproductive dysfunction in livestock.

These findings advance our understanding of heat stress pathophysiology in male reproduction and highlight metabolic pathways as promising intervention targets. Future research should assess the in vivo efficacy of melatonin and explore its use in breeding strategies to improve thermotolerance in goats and other livestock species.

## 4. Materials and Methods

### 4.1. Animals and Ethics Statement

All animal procedures were performed in accordance with the National Standards for the Care and Use of Laboratory Animals in China and were approved by the Animal Welfare and Ethical Committee of China Agricultural University (Approval No. AW13014202-1-11, 31 October 2024).

### 4.2. Isolation, Purification, and Identification of SCs

SCs were isolated from the testes of 4-month-old Hainan black goats via a two-step enzymatic digestion. Briefly, minced testicular tissues were first digested with 4 mg/mL collagenase IV (Invitrogen, Carlsbad, CA, USA) and 0.2% hyaluronidase (MedChemExpress, Shanghai, China), followed by digestion with 0.25% trypsin (Invitrogen, NY, USA) and 10 μg/mL DNase I (Invitrogen, NY, USA) at 37 °C. The resulting cell suspension was filtered, centrifuged, and resuspended in DMEM/F12 medium (Invitrogen, NY, USA) supplemented with 10% FBS (Thermo Fisher Scientific, Carlsbad, CA, USA) and 1% penicillin-streptomycin. Cell purification was achieved by differential plating, replacing the medium after 6 h of culture to remove non-adherent cells. SCs were verified by immunofluorescence staining using an anti-SOX9 antibody (1:200; Beyotime Biotechnology, Shanghai, China), with nuclei counterstained using DAPI (Beyotime Biotechnology, Shanghai, China), and visualized under a fluorescence microscope.

### 4.3. Cell Viability Assay

Cell viability was assessed using a Cell Counting Kit 8 (Beyotime Biotechnology, Shanghai, China). SCs were plated at 1 × 10^4^ cells/well in 96-well plates and allowed to adhere for 8 h. To test the direct effect of melatonin, cells were treated with different concentrations of melatonin (0.05, 0.5, and 1 µM) in 0.1% DMSO for 24 h, with a control group receiving 0.1% DMSO alone. For the heat stress experiment, cells were pretreated with melatonin for 1 h prior to the onset of heat stress; the corresponding control was treated with 0.1% DMSO and maintained at 37 °C. After treatment, 10 µL of CCK-8 solution was added to each well, followed by 2 h of incubation at 37 °C. Absorbance was measured at 450 nm using a Bio-Rad microplate reader (Bio-Rad Laboratories, Hercules, CA, USA). Each condition was tested in five replicates, and viability was expressed as a percentage relative to the control.

### 4.4. RNA-seq Analysis

RNA-seq analysis was performed using the BGISEQ-T7 platform on samples from three groups: control (Control 1, Control 2, Control 3) and heat stress (HS 1, HS 2, HS 3), both treated with 0.1% DMSO, and a heat stress with melatonin group (HS + Melatonin 1, HS + Melatonin 2, HS + Melatonin 3) supplemented with 0.5 µM melatonin in the same DMSO concentration. After quality control and filtering, clean reads were aligned to the reference genome using HISAT2. Gene annotation was performed using multiple databases including Nr, Nt, Pfam, KOG/COG, Swiss Prot, KEGG Orthology (KO), and GO. The transcriptome data have been deposited in the NCBI Sequence Read Archive (SRA) under accession number PRJNA1347640. Gene expression was quantified by Fragments Per Kilobase of transcript per Million mapped reads (FPKM), and differential expression analysis was consequently performed using DESeq2 (FDR < 0.05 and |log_2_ (fold change)| ≥1). Enrichment analyses of GO terms and KEGG pathways were performed using GOseq and KOBAS, respectively.

### 4.5. RT-qPCR

Total RNA (1 μg) isolated from SCs was subjected to reverse transcription using the Swe-Script All-in-One RT SuperMix for qPCR (Servicebio, Wuhan, China). The resulting cDNA was then amplified by qPCR on an ABI QuantStudio 3 system (Applied Biosystems, Santa Clara, CA, USA). Each 20 μL reaction comprised 10 μL of 2× Universal Blue SYBR Green qPCR Master Mix (Servicebio, Wuhan, China), 0.8 μL each of forward and reverse primers (10 μM), 1 μL of cDNA template, and 7.4 μL of RNase-free H_2_O. Gene expression levels were calculated via the 2^−ΔΔCt^ method, with *GAPDH* serving as the endogenous control. All primer sequences are detailed in Supplementary Table S1.

### 4.6. Transmission Electron Microscopy

For TEM analysis, SCs were primarily fixed with TEM fixative at 4 °C for 4 h. After being washed with 0.1 M phosphate buffer (PB, pH 7.4), the samples were pre-embedded in warm 1% agarose solution. The agarose blocks were then post-fixed in 1% osmium tetroxide (in 0.1 M PB) for 2 h at room temperature in the dark, followed by additional PB washes. Dehydration was carried out using a graded ethanol series (30% to 100%) and 100% acetone. The samples were subsequently infiltrated and embedded with EMBed-812 resin, and polymerized at 60 °C for 48 h. Ultrathin sections (60–80 nm) were cut using an ultramicrotome, collected on 150-mesh copper grids, and double-stained with uranyl acetate and lead citrate. The grids were finally observed under a TEM for image acquisition.

### 4.7. Measurement of Mitochondrial Membrane Potential

The mitochondrial membrane potential was assessed using the JC-1 Mitochondrial Membrane Potential Assay Kit (Servicebio, Wuhan, China) according to the manufacturer’s instructions. Briefly, cells were incubated with JC-1 staining working solution at 37 °C for 20 min in the dark. After washing with staining buffer, cells were imaged under a fluorescence microscope. The red-to-green fluorescence ratio was quantified from multiple random fields to determine the extent of mitochondrial depolarization.

### 4.8. Measurement of Mitochondrial Superoxide by Flow Cytometry

Mitochondrial superoxide levels were measured using MitoSOX™ Red reagent (MedChemExpress, Shanghai, China) followed by flow cytometric analysis. Cells were adjusted to a density of 1 × 10^6^ cells/mL, washed with PBS, and incubated with 5 μM MitoSOX™ Red working solution (prepared from a 5 mM stock in serum-free medium) at 37 °C for 30 min in the dark. After incubation, cells were washed twice with PBS and resuspended in phenol red free DMEM medium. Fluorescence intensity was analyzed immediately using a flow cytometer (excitation/emission: 510/580 nm), with data from at least 10,000 events collected per sample. Mean fluorescence intensity (MFI) was quantified using FlowJo software (Version 10.10.0, BD Biosciences, Ashland, OH, USA).

### 4.9. Metabolic Flux Analysis

Prior to heat stress, SCs were incubated for 24 h in medium containing 25 mM [U-^13^C_6_] glucose (MedChemExpress, Shanghai, China) and 10% FBS (Thermo Fisher Scientific; Life Technologies Corp, Carlsbad, CA, USA), and melatonin (for the HS + Melatonin group). This 24-h labeling and pretreatment period was selected as it is sufficient to achieve isotopic steady-state labeling of glycolytic and TCA cycle intermediates, as demonstrated in previous studies [54,55]. Following this labeling period, heat stress was applied to the HS and HS + Melatonin groups by transferring the cells to a 42 °C incubator for 0.5 h. The Control group was maintained at 37 °C throughout the entire experiment. Cells remained in the [U-^13^C_6_] glucose medium during the 0.5 h heat stress.

Metabolite extraction was performed as described [56,57]. Briefly, cells were washed with ice cold saline and quenched with 500 μL of −20 °C methanol. After adding internal standards (norvaline and D_31_-palmitate), cells were scraped and extracted in a chloroform/methanol/water mixture on ice. The aqueous and organic phases were separated by centrifugation (12,000× *g*, 10 min), collected, and vacuum-dried.

For gas chromatography-mass spectrometry (GC-MS) analysis, dried aqueous phase metabolites were derivatized via methoximation and silylation. The organic phase was transesterified to yield fatty acid methyl esters (FAMEs). Metabolite separation and detection were performed using a GC-MS system (Thermo Trace 1300/ISQ) with a DB-5 column, following established methods [56,57].

MIDs were determined from raw GC-MS data using the INCA software(Version 4.1), with correction for natural isotope abundance. The contribution of [U-^13^C_6_] glucose to biosynthesis was assessed using Isotopomer Spectral Analysis (ISA). Molar percent enrichment (MPE) was calculated as previously reported [56,57].

### 4.10. Measurement of Mitochondrial Respiration

SCs (1 × 10^4^ cells/well) from control, HS, and HS + Melatonin groups were seeded in a 96-well XFe plate and incubated overnight (*n* = 3). The HS + Melatonin group was pretreated with 0.5 μM melatonin for 1 h before heat stress. Both HS and HS + Melatonin groups then underwent heat stress at 42 °C for 0.5 h. Mitochondrial respiration was analyzed using a Seahorse XFe 96 (Agilent Technologies, Santa Clara, CA, USA) Analyzer. Following overnight hydration of the sensor cartridge at room temperature, ports were loaded with: A, 20 μL of 1.0 μM oligomycin; B, 22 μL of 0.5 μM FCCP; C, 25 μL of 0.5 μM rotenone/antimycin A. Cells were changed to XF Base medium containing 10 mM glucose, 1 mM pyruvate, and 2 mM glutamine. After calibration, the plate was assayed over three measurement cycles. Data were normalized to protein content and analyzed with Wave software (Version 1.0.5).

### 4.11. Statistical Analysis

Data are presented as mean ± SD from biological replicates (*n* indicated in each figure). Statistical comparisons for three or more groups were performed using one-way ANOVA followed by Tukey’s test, while comparisons between two groups were conducted using Welch’s *t*-test.

## 5. Conclusions

In conclusion, our integrated transcriptomic and metabolic flux analysis confirms that acute heat stress triggers profound metabolic disruption in goat Sertoli cells, primarily by suppressing mitochondrial glucose oxidation. Strikingly, melatonin-mediated protection involves a metabolic reprogramming that further attenuates this glucose carbon flux while simultaneously restoring mitochondrial bioenergetics. This apparent paradox points to a compensatory shift toward alternative fuels, highlighting melatonin’s role as a metabolic regulator that promotes cellular resilience by enhancing metabolic flexibility. These insights underscore the primacy of metabolic dysregulation in heat-induced testicular injury and provide a strong rationale for exploring the therapeutic application of melatonin in livestock production under climate stress.

## 6. Patents

The authors declare that there are no patents resulting from the work reported in this manuscript.

## Data Availability

The raw transcriptome data generated during this study are openly available in the NCBI database, Sequence Read Archive (SRA), under accession number PRJNA1347640.

## Supplementary Materials

The following supporting information can be downloaded at: https://www.mdpi.com/article/10.3390/ijms262311475/s1.
