# Supplementary material for: Melatonin Rescues Heat Stress-Induced Suppression of TCA Cycle and Mitochondrial Damage in Goat Sertoli Cells
Source: Int J Mol Sci. 2025 Nov 27;26(23):11475. doi: 10.3390/ijms262311475 (PMC12692276; doi:10.3390/ijms262311475)
Supplement: Supplementary file 1 [file ijms-26-11475-s001.zip › Supplementary Materials.pdf]

Table S1. The primer sequences used for qRT-PCR (F: forward, R: reverse)

Gene		Primer Sequence (5'-3')
HSPA6	*F*	AAGGAGACGGCGGAGGCTTAC
	R	GCTGCGAGTCGTTGAAGTAGGC
HSPA4L	*F*	GACCTCGGCTTTCTCAACTGCTAC
	R	CACCTGTCGCTGTACTCATTGGC
CCNF	*F*	CGGGGAACCTGAAGCTCTTT
	R	TCAGACACCGACAAGCCTTC
HDHD3	*F*	TCCTGAAAGGTGTTGGCCTG
	R	ATGGAAAATACGGGGGTCGG
PTGS2	*F*	TGGTCTGGTGCCTGGTCTGATG
	R	TGTCTGGAACAACTGCTCATCGC
FREM3	*F*	ACACTGAACTTCGCTCCAGG
	R	TCAAACCGCTCTGAACCCTC
MYOC	*F*	GGAATGAAGAGGGAGGCACTG
	R	CTCATCCACACGCCGTACTT
FLT1	*F*	AAGCTGAGGAGAAGGGCAAC
	R	GAGAGGGGGTTTTCACTCCG
E2F8	*F*	ATCATTTGCAGGGACAGGGG
	R	TGGAGCCTGTTGGGTTTGTT
LRRC8E	*F*	CAAAGGTTTGTCTTTGGGCGG
	R	GTGAAATCTGGCAATGGGCG
